# From forest and agro-ecosystems to the microecosystems of the human body: what can landscape ecology tell us about tumor growth, metastasis, and treatment options?

**DOI:** 10.1111/eva.12031

**Published:** 2012-11-22

**Authors:** Simon P Daoust, Lenore Fahrig, Amanda E Martin, Frédéric Thomas

**Affiliations:** 1 M.I.V.E.G.E.C (Maladies Infectieuses et Vecteurs: Ecologie, Génétique, Evolution et Contrôle) UMR (IRD/CNRS/UM) 5290, Centre IRD- 911 Avenue Agropolis – B.P. 64501Montpellier, France; 2 Geomatics and Landscape Ecology Research Laboratory, Biology Department, Carleton UniversityOttawa, ON, Canada

**Keywords:** cancer ecology, evolutionary medicine, landscape ecology, tissue microhabitat landscape

## Abstract

Cancer is now understood to be a process that follows Darwinian evolution. Heterogeneous populations of cancerous cells that make up the tumor inhabit the tissue ‘microenvironment’, where ecological interactions analogous to predation and competition for resources drive the somatic evolution of cancer. The tumor microenvironment plays a crucial role in the tumor genesis, development, and metastasis processes, as it creates the microenvironmental selection forces that ultimately determine the cellular characteristics that result in the greatest fitness. Here, we explore and offer new insights into the spatial aspects of tumor–microenvironment interactions through the application of landscape ecology theory to tumor growth and metastasis within the tissue microhabitat. We argue that small tissue microhabitats in combination with the spatial distribution of resources within these habitats could be important selective forces driving tumor invasiveness. We also contend that the compositional and configurational heterogeneity of components in the tissue microhabitat do not only influence resource availability and functional connectivity but also play a crucial role in facilitating metastasis and may serve to explain, at least in part, tissue tropism in certain cancers. This novel work provides a compelling argument for the necessity of taking into account the structure of the tissue microhabitat when investigating tumor progression.

## Introduction

Although our understanding of cancer biology and genetics has greatly improved since Richard Nixon's 1970s call to arms against cancer, the treatments developed have not lived up to the expectations (Jemal et al. 2009; Ryan et al. 2010; Colotta 2011; Drake 2011). For this reason, there has been a growing need for a shift in the way cancer is traditionally studied and treated (Merlo et al. 2006; Pienta et al. 2008; Aktipis et al. 2011). It is within this context that a fundamentally different approach to cancer research has emerged: the study of cancer as a process following Darwinian evolution (Cairns 1975; Nowell 1976; Crespi and Summers 2005; Merlo et al. 2006). This Darwinian framework provided a novel paradigm that allowed researchers to use evolutionary theory to elucidate mechanisms that drive natural selection among cancerous cells within the tumor (Crespi and Summers 2005; Pienta et al. 2008; Gatenby et al. 2010; Colotta 2011). Here, genetically and epigenetically heterogeneous populations of cancerous cells that make up the tumor are described as inhabiting the tissue ‘microenvironment’, where ecological interactions such as predation and competition for resources drive the somatic evolution of cancer (Crespi and Summers 2005; Pienta et al. 2008; Bozic et al. 2010; Marusyk and Polyak 2010; Greaves and Maley 2012).

Several interdisciplinary studies that used or inferred from well-established ecological models to predict cancer growth or metastasis arose from this conceptual breakthrough. For instance, González-García et al. (2002) used classic metapopulation models designed to predict population persistence within a patchy habitat to study the dynamics of tumor heterogeneity; Gatenby et al. (2009a, b) developed novel cancer treatment protocols by adopting techniques used in the control of invasive species; Marco et al. (2009) drew on the similarities between tumor metastasis and long-distance dispersal by plants to develop predictive models for tumor metastasis; and finally, Ryan et al. (2010) compared tumor growth and metastasis to suburban sprawl development. A finding that was consistent in all these interdisciplinary studies, and also confirmed by recent molecular, genomic, and modeling works (Chung et al. 2005; Anderson et al. 2006; Castelló-Cros et al. 2009; Polyak et al. 2009; Egeblad et al. 2010; Lee et al. 2011), was the crucial role played by the tumor microenvironment in the processes of tumor genesis, development, and metastasis. While tumor cells are continuously evolving through cumulative genetic and epigenetic changes, it is the selection forces in the microenvironment that ultimately determine the cellular characteristics that will result in the greatest fitness (Hanahan and Weinberg 2011). This is exemplified by the fact that not only tumor growth, invasiveness, and metastasis were influenced by resource availability, the former were also shown to be greatly affected by the spatial arrangement of noncancerous cells and macromolecules in the tumor microenvironment (Anderson et al. 2006; Lee et al. 2011). A heterogeneous spatial arrangement drives selection toward a few dominant clones, with a high propensity to emigrate from the tumor (metastasis), with invasive (fingering margins) tumor morphology, whereas homogeneous spatial arrangements allow for the coexistence of many phenotypes, more or less aggressive, with noninvasive (smooth margins) tumor morphology (Anderson et al. 2006; Chen et al. 2011; Lee et al. 2011).

Here, we explore and offer new insights into the spatial aspects of tumor–microenvironment interactions by comparing landscape ecology theory with tumor growth and metastasis within the tissue microhabitat, an approach that has already successfully applied to health problems such as antibiotic resistances in wildlife (Singer et al. 2006). Landscape ecology provides the ideal framework for this task as it explicitly addresses the effects of composition and spatial configuration of mosaics (grouping of discrete patches) on a wide range of ecological responses (e.g., abundance and distribution of organisms) (Wiens et al. 1993). The underlying premise of landscape ecology is that the composition and configuration of a landscape mosaic affect ecological systems in ways that would be different if the mosaic composition or configuration were different (Wiens 1995). First, we define certain key concepts of landscape ecology theory and then draw parallels between the various constituents of the tissue microhabitat, the tumor, and the tumor development process to these concepts. We then briefly highlight similarities between processes that govern plant and animal population growth, spread, and long-distance dispersal in heterogeneous landscapes with those of tumor growth, local invasion, and metastasis in the tissue microhabitat to provide novel perspectives on the spatial mechanisms that govern the latter. Lastly, we take advantage of insights obtained from research documenting the impacts of agricultural intensification on biodiversity to comment on some of the newly proposed avenues of cancer therapy.

## Defining the tumor microhabitat landscape and the ecology of the tumor and tumor development process

An important goal of landscape ecology is to understand how landscape structure affects population dynamics (e.g., births, deaths, movement, species interactions, etc.) and the resulting abundance and distribution of organisms (Fahrig 1999). Although most people have an intuitive understanding of what is meant by landscape, landscape structure, and population dynamics, we will, nonetheless, define these and other commonly used terms, as they are the conceptual foundations from which we will build (see Table 1 for complete list of definitions).

**Table 1:** Definitions of common terms used in ecology and landscape ecology and their relevance to tumor landscape ecology. *Idem* indicates that the term, as defined in ecology and/or landscape ecology, does not differ as applied to cancer ecology

Terms	Defined within ecology or landscape ecology	Applied to cancer ecology
Carrying capacity	Population size that can be sustained over the long term within a given area.	Maximum tumor size given the resources and space available within the local tissue microhabitat.
Community	Group of interacting species within a given area.	Assemblage of two or more genotypically and phenotypically distinct cancer cell populations within the tumor.
Cover type	A categorical classification of landscape features, based on a set of observable characteristics, for example, vegetation type.	A categorical classification of cell types, based on a set of observable characteristics.
Ecosystem disturbance	An event that disrupts an ecosystem sufficiently to change its functioning, usually involves removal of biomass.	Rapid drop in local resources (glucose, oxygen, etc.), chemotherapy, physical trauma or damage to tissue, etc.
Functional connectivity	Degree to which the landscape facilitates movement of the species or species group among its habitat patches	Degree to which the tissue stroma facilitates movement of cancer cells.
Grain size	Average size (diameter or area) of the patches in a landscape; a ‘coarse-grained’ landscape contains large patches.	*Idem*
Habitat	Area that is inhabited by a particular species of animal, plant, or other type of organism.	Tissue microhabitat
Habitat patch	A discrete area of habitat of the species/species group.	A discrete area of habitat of the cancer cell population/group of populations.
Landscape	Area that is spatially heterogeneous in at least one factor of interest.	Spatial distribution of the components within and surrounding the local tissue microhabitat.
Landscape composition	The cover types present in a landscape, and the amounts of each.	Amount of each cell and macromolecule type within the tissue microhabitat.
Landscape configuration	Spatial distribution of the cover types in a landscape.	Spatial distribution of each cell and macromolecule type within the tissue microhabitat.
Landscape heterogeneity	Diversity and pattern complexity of cover types in the landscape; a landscape with more cover types in a more complex configuration is more heterogeneous.	Complexity of the tissue microhabitat in both composition and configuration of components.
Landscape matrix	Nonhabitat part of the landscape (which could be comprised of several cover types).	*Idem*
Landscape structure	Composition, configuration, and heterogeneity of cover types.	Composition, configuration, and heterogeneity of noncancerous cells, extracellular matrix, and physical parameters such as gradients of oxygen, glucose, pH, and interstitial pressure
Long-distance dispersal (LDD)	Movement of animals, plants, or other organisms to new, usually separate habitats.	Metastasis
Population	A discrete group of individuals of the same species, where interactions among individuals in a population are much more frequent than interactions among individuals in different populations.	Group or cluster of cancer cells within a tumor that are similar enough in genotype to have nearly indistinguishable phenotypes.
Population growth	Increase in the number of individuals in a population over time.	Tumor growth
Scale	Spatial or temporal dimension of an object or process, characterized by both grain and extent.	*Idem*
Spatially explicit metapopulation model	Population model in which the population is divided into discrete subpopulations, where dispersal among subpopulations depends on their spatial relationships.	*Idem*
Spread/Invasion	Expansion of a species into a local area not previously occupied by it.	Tumor invasion of local tissue.

### Tissue microhabitat landscape and structure

As noted by Turner et al. (2001), landscapes are generally thought of as expanses of land and water that can be observed from a vantage point and are therefore subject to the perspective of the ‘observer’, that is, organism or group of organisms. If we consider the fact that most organisms experience/view their surroundings at very different scales (e.g., cancer cells versus small wasps versus migratory birds) (Daoust et al. in press), a general definition of what constitutes a landscape must therefore be broad enough to encompass any area at any relevant scale. Thus, a landscape is broadly defined as an area that is spatially heterogeneous in at least one factor of interest.

Tumors, as approached from an ecological perspective, can be seen as populations of cancerous cells that inhabit a noncancerous, organotypic tissue microhabitat (De Wever and Mareel 2003; Chung et al. 2005; Castelló-Cros et al. 2009; Marusyk and Polyak 2010; Lee et al. 2011). This heterogeneous microhabitat consists of many components including noncancerous cells (endothelial cells, pericytes, smooth-muscle cells, fibroblasts, etc.), extracellular matrix (ECM), and physical parameters such as gradients of oxygen, glucose, pH, and interstitial pressure (Chung et al. 2005; Merlo et al. 2006; Lee et al. 2011). Furthermore, the tissue microhabitat is embedded within an organ and organ complex within which molecules (growth factors, metalloproteinases and/or angiogenic molecules, etc.) diffuse readily (Chung et al. 2005). The landscape context of the tumor microhabitat can be seen as the spatial distribution of components within and immediately surrounding the tissue microhabitat.

The landscape structure of the tissue microhabitat can be characterized by the composition, configuration, and heterogeneity of the various components listed above. Briefly, landscape composition describes the types and amounts of different components, whereas its configuration refers to their spatial arrangement within the microhabitat landscape. Within terrestrial systems, landscape composition has strong, direct effects on population dynamics and persistence through its effects on reproduction and mortality, whereas landscape configuration affects population dynamics indirectly through its effects on interpatch movement (Fahrig and Nuttle 2005).

### Ecology of the tumor and tumor developmental process

Ecologists study the dynamics of communities of species and their interactions with each other and their environment (Merlo et al. 2006). When applying an ecological perspective to human cancers, populations of cancer cells (i.e., tumor clones) that differ in heritable traits (genotypically heterogeneous) can be considered distinct ‘species’ within the tumor. Together, these populations make up the cancer cell community that forms the tumor (Marusyk and Polyak 2010). The coexistence of phenotypically distinct populations of tumor cells should inevitably lead to the formation of a network of biological interactions that drives selection (Crespi and Summers 2005; Marusyk and Polyak 2010). Some of the key interactions that are likely to exist between distinct tumor clones are competition, antagonism, and mutualism (Marusyk and Polyak 2010).

Another important aspect of tumor ecology is the potential of cancer cells to move into local or distant tissue microhabitats. Here, local tissue invasion by cancer cells can be compared with the spread of animal or plant populations within a contiguous habitat, whereas cancer cell metastasis is generally accepted to be closely comparable with long-distance dispersal (LDD) as well as invasion and spread of exotic species (Table 1) (Gatenby et al. 2009a, b; Marco et al. 2009).

## Effect of the tissue microhabitat landscape on tumor growth, local invasion, and metastasis

Empirical and theoretical research on plant and animal models have clearly established that resource availability and distribution within a habitat and its landscape context can significantly impact the density, spread, and long-distance dispersal of populations (MacArthur and Levins 1964; MacArthur and Pianka 1966; MacArthur and Wilson 1967; Bender et al. 1998; Fahrig 2003, 2007; Fahrig and Nuttle 2005; Foley et al. 2005; Bacles et al. 2006; Coutts et al. 2011; Minor and Gardner 2011; North et al. 2011). Although the scale and environmental context of plant and animal populations differ significantly from those of cancer cells, enough similarities exist among them (Crespi and Summers 2005; Merlo et al. 2006; Gatenby 2009; Marco et al. 2009) that observations made on the former models can serve, by analogy, to explain and even predict the responses of the latter to landscape structure.

### Size of the tissue microhabitat

In classical island biography theory (MacArthur and Wilson 1967), larger habitats (or patches) contain more resources and therefore have larger carrying capacities than smaller ones, leading to higher species richness and abundance. Similar constraints also exist for cancer cell populations as primary tumors develop in tissue microhabitats with varying resources and architectural constraints or barriers (Greaves and Maley 2012). In some instances, tumors could potentially develop in high-quality tissue microhabitats (low nutritional or spatial constraints), resulting in little competition between cancerous cells and increased tumor growth. Conversely, large spatial/architectural and resource constraints could increase significantly the competition between cancer cells, limiting the size of the primary tumor. To our knowledge, this idea remains unexplored.

In addition to influencing population (or tumor) size, habitat area can also be an important force driving local and long-distance population dispersal (North et al. 2011). Animals disperse for many reasons but mainly to avoid intraspecific competition, particularly kin competition, in the current site, and to take advantage of exploitable resources elsewhere (Fahrig 2007). The larger and more rich resources the habitat is, the lower the probability that an individual will leave it, due to increased energy expenditure and higher mortality risk; therefore, it is plausible to expect that organisms should move only when the costs of remaining outweigh the potential costs of leaving the habitat (North et al. 2011). Cancer cells within the tissue microhabitat are faced with the same trade-off (Polyak et al. 2009; Marusyk and Polyak 2010; Lee et al. 2011). Unlike animal models, cancer cells do not ‘decide’ to leave the primary tumor habitat. That being said, recent evidence has shown that, under poor conditions, selection favors highly mobile, invasive, and metastatic cancer phenotypes (Anderson et al. 2006; Lee et al. 2011). Therefore, the size of the tissue microhabitat where the primary tumor developed is an important factor driving the selection for more aggressive, invasive, and metastatic phenotypes.

Habitat size can also affect populations by influencing the proportional amounts of edge and interior habitats (Bender et al. 1998; Fagan et al. 1999). As previously discussed, species within natural communities often compete for space and resources. One way to minimize competition between coexisting species that use similar resources may be to partition these resources in space or time to reduce niche overlap (Pianka 1974; Pianka and May 1981). This can give rise to different habitat or ‘niche’ specialists; for example, edge species are those associated primarily with the perimeter of a habitat patch, while interior species are those associated with the center of patches (Hayden et al. 1985; Freemark and collins 1992; Askins 1995; Bender et al. 1998). In a meta-analysis exploring the effect of habitat patch size on population density, Bender et al. (1998) revealed that populations of interior species decline as patch size decreases, whereas the opposite effect is seen in edge species, whose population densities increase as patches become smaller and the proportional amounts of edge habitat increases. Edge species are often more likely to disperse to other habitats than interior species; for example, 90% of Costa Rican migrant bird species are found inhabiting forest and canopy edges (Levey and Stiles 1992). This increased propensity to disperse in edge species may result from their association with the greater compositional and structural complexity usually found at boundaries (Imbeau et al. 2003). Therefore, smaller habitats favor edge species that are more likely to disperse. Similarly, resource partitioning and niche segregation were proposed as one of the mechanisms involved in maintaining tumor heterogeneity (analogous to species richness) (Nagy 2004; Marusyk and Polyak 2010; Durrett et al. 2011). Here, cancer cells found on the periphery of the tumor (edge specialist) are exposed to, and shaped by, the tissue microenvironment as opposed to those inside the tumor (Marusyk and Polyak 2010). Interactions between tumor cells and microenvironment have been shown to both shape malignant behavior and promote tumor progression (Park et al. 2000; Liotta et al. 2001; Marusyk and Polyak 2010). In line with animal models in terrestrial habitats, small tissue microhabitats could lead to higher cancer cells exposed to the microenvironment, potentially leading to the selection of more aggressive and invasive cancer cells.

### Heterogeneity of the tissue microhabitat landscape

Since the groundbreaking work of MacArthur (MacArthur and Levins 1964; MacArthur and Pianka 1966), ecologists have been studying the impact of the spatial distribution of resources on population dynamics as they can act as a strong selective force, shaping animal life-history traits and behaviors (Bolhuis and Giraldeau 2005). Spatio-temporally variable landscapes may drive selection of traits that promote dispersal, as greater dispersal abilities and propensity to disperse in these landscapes may confer fitness advantages, allowing individuals and populations to track available resources and escape declining local conditions. Indeed, animals that rely primarily on resources that are spatially or temporally heterogeneous (predators, parasitoids, etc.) are generally highly mobile and disperse readily, allowing them to exploit several prey populations or resource patches (Tscharntke and Brandl 2004; Frair et al. 2005; Roshier et al. 2008). It has also been suggested that dispersal may act as a ‘bet-hedging’ strategy (Den Boer 1968), and genotypes that result in higher dispersal abilities are likely to have greater probabilities of persistence in the landscape, as declines in local conditions or local extinctions are less likely to result in the loss of this genotype from the population (e.g., Friedenberg 2003). Interestingly, it would also appear that cancer cells respond similarly to spatial heterogeneity in resource availability. Indeed, using a multi-scale mathematical model of cancer invasion, Anderson et al. (2006) reported that simulated tumors were much more invasive and aggressive in structurally heterogeneous environments. Although the authors attributed the selection for more invasive and aggressive phenotypes to lower resource availability, the spatial distribution of resources could have also been an important selective force driving invasiveness.

Another important landscape component that can influence the degree to which species or species groups move among habitat patches (functional connectivity) is the composition and configuration of the landscape matrix (Goodwin and Fahrig 2002; Bender et al. 2003; Tischendorf et al. 2003; Bender and Fahrig 2005). Indeed, the surrounding matrix of a habitat fragment is typically not homogeneous: it may contain patches of various cover types, for example, agricultural fields, housing, etc., which could affect the connectivity among habitat patches and potentially influence meta- and local population structure and dynamics (Marshall and Moonen 2002; Steffan-Dewenter 2003; Fahrig 2007). Through both computer simulations and empirical observations, Bender and Fahrig (2005) found that habitat patch size and isolation are poor predictors of interpatch movement when the landscape matrix contains many different cover types (heterogeneous matrix) in a coarse-grained pattern. These authors suggest that features in the matrix influence interpatch animal movement rates, for example, they avoid cover types that are perceived to be inhospitable or impermeable by going around them (Bender and Fahrig 2005). Although cancer cells can engineer the microhabitat around them, making it more hospitable (Castelló-Cros et al. 2009), the composition and arrangement of macromolecules within the extracellular matrix have been shown to influence tumor growth and invasiveness (De Wever and Mareel 2003; Anderson 2005; Castelló-Cros et al. 2009). Indeed, the spatial distribution of molecules involved in cell adhesion and motility (laminin, fibronectin, and vitronectin) can facilitate local tissue invasion as well as intravasation (locally invasive carcinoma cells entering into the lumina of lymphatic or blood vessels) (Anderson 2005; Valastyan and Weinberg 2011).

A unique property of cancer cells is their ability to mimic the gene expression profiles of noncancerous cell types found in their environment (Chung et al. 2005; Pienta et al. 2008). In other words, cancer cells can acquire the characteristics of noncancerous cells that are close by. Therefore, the compositional heterogeneity of the tissue microhabitat (proportion of different noncancerous cell types found within the local microhabitat) can also have a dramatic effect on tumor progression (Kenny and Bissell 2003; Merlo et al. 2006). The best documented example of this effect is that of prostate cancer bone metastasis (reviewed in Chung et al. 2005). In their review, Chung et al. (2005) showed that genetic alterations in prostate cancer cells alone are not enough to confer metastatic status without a supporting tumor microenvironment. The authors report that by acquiring the characteristics of other cell types, such as osteoblasts and osteoclasts, cancer cells are able to metastasize to distant bone and visceral organs (tissue tropism) (Chung et al. 2005). Thus, the compositional and configurational heterogeneity of components in the tissue microhabitat do not only influence resource availability and functional connectivity but also play a crucial role in facilitating metastasis and may serve to explain, at least in part, tissue tropism in certain cancers.

### Disturbances within the tissue microhabitat

Habitat fragmentation can be characterized as a ‘landscape-level’ disturbance, which is defined as a break or gap in the habitat or landscape structure continuum (Bergelson et al. 1993; With 2002). Such disturbances are unanimously acknowledged to influence the spread of invasive plants (Bergelson et al. 1993; With 2002; Hastings et al. 2005; Meulebrouck et al. 2009), as such breaks and gaps in fact serve as habitats for these invasive plants, which is why they are so commonly observed in disturbed areas such as roadsides (Amor and Stevens 1976; Gelbard and Harrison 2003; Switalski et al. 2004) and in grazed or cultivated fields (Sawada et al. 1982; Bergelson et al. 1993). It is therefore not surprising that size and distance between ‘gaps’ in the habitat continuum have also been shown to influence invasion, as these would be increasing the amount of habitat for these species. Bergelson et al. (1993) showed that the rate of spread of an invasive grass was sensitive to both the gap size and the gap distribution; the invasive weed *Senecio vulgaris* moved a greater distance within the habitat when the gaps were large and closer together (Bergelson et al. 1993). Interestingly, invasion of new tissue habitats by metastasizing cancer cells has also been shown to respond positively to habitat availability generated specifically by disturbances (De Wever and Mareel 2003; Marco et al. 2009). Indeed, tissue invasion by cancers appears to be greatly facilitated by tissue damage or lesions; cancer invasion is stimulated by wounding of the host tissue as shown by rat colon adenocarcinoma cells that were transplanted into experimentally induced subcutaneous granulation tissue and in undisturbed subcutaneous tissue (Mareel et al. 1991; De Wever and Mareel 2003). Hence, one must also take into consideration the presence, size, and distribution of breaks (lesions or damage) within the tissue microhabitat landscape, as these are also important to the metastatic and tissue invasion processes.

## Lessons from agricultural intensification and species loss applied to cancer treatments

Ecological and evolutionary theory has not only improved our understanding of cancer progression but has also led to the development of several novel cancer therapies (Pienta et al. 2008; Gatenby 2009; Gatenby et al. 2009b). One such treatment option suggests that an efficient way to kill cancer cells may be to modify or target the tissue microhabitat (stromal therapy), rendering it inhospitable to the multiplying cancer cells (De Wever and Mareel 2003; Pienta et al. 2008; Greaves and Maley 2012). As expressed by Pienta et al. (2008) ‘often, the most efficient way to kill a species is to destroy its niche by altering the environment’. This is an interesting proposal, but it has one significant flaw; it is founded on the assumption that we can target and eliminate or alter all potential niches for the different cancer phenotypes within the habitat. Unfortunately, this might not be possible. The recent and major changes to the agricultural landscape, known as agricultural intensification, provide an ideal framework to address this issue (Robinson and Sutherland 2002).

Very briefly, agricultural intensification is characterized by the transformation of heterogeneous, complex landscapes (extensive farmlands) to homogenous, simple landscapes containing only fragments of natural or semi-natural land (intensive farmlands) (Matson et al. 1997). This process is considered to be one of the major drivers of species loss worldwide (Benton et al. 2003; Green et al. 2005; Wilson et al. 2005). Although the majority of species studied to date are negatively affected by agricultural intensification (Burel et al. 1998; Donald et al. 2001; Benton et al. 2002; Fahrig 2003; Wickramasinghe et al. 2004; Bàrberi et al. 2010), some, however, are actually shown to benefit from it (Burel et al. 2004; Flohre et al. 2011; Ragsdale et al. 2011). In a study comparing the response of various taxa to agricultural intensification, Burel et al. (2004) reported that, although the total number of beetle species remains the same between intensive and extensive farmlands, the species composition is drastically different. Here, larger beetle species that inhabit forested habitats within extensive farmlands are substituted for smaller, highly dispersive species adapted to high rates of disturbance (Burel et al. 2004). By analogy, unless all potential niches in the habitat are altered or eliminated, similar outcomes could be expected for the impact of stromal therapy on the community of cancerous cells forming the tumor. Then again, one could argue that, in theory, stromal therapy could work by ensuring that the only niches left are those selecting for less aggressive phenotypes. Regardless, an important problem with stromal therapy, as we have already shown, is that disturbances or changes in the structure of the tissue microhabitat can have significant effects on tumor growth and metastasis, and this not always to the advantage of the patient. For this reason, unless we significantly improve our understanding of the role played by the tissue microhabitat in governing tumor growth, we are forced to agree with Polyak et al. (2009) that the side effects of ‘stromal therapy’ could be serious and difficult to predict.

## Conclusion

Although previous studies have clearly shown that ecological interactions such as competition, mutualism, and antagonism are likely to shape somatic evolution of cancer cells (Crespi and Summers 2005; Marusyk and Polyak 2010), up until now, no study has placed these interactions within a spatial context. Cancerous cell populations that form the tumor community inhabit a heterogeneous tissue microhabitat that has an important role in the tumor development process. By placing tumors within a tissue microhabitat landscape perspective, we were able to make analogies with examples drawn from both theoretical and empirical landscape ecology studies. We show that, as predicted by theory and in line with animal models, both the size and spatial pattern (composition and configuration) of the tissue microhabitat can drive selection toward more aggressive, invasive, and dispersive phenotypes. This novel work provides a compelling argument for the necessity of taking into account the structure of the tissue microhabitat when investigating tumor progression.

A recent and major objective for landscape ecologists has been to enhance biodiversity within agro-ecosystems through the alteration of landscape patterns without lowering agricultural productivity (Fahrig et al. 2011). With this task in mind, Fahrig et al. (2011) developed a new framework for the study of population abundance and distribution within heterogeneous landscapes. They replaced the classic ‘structural landscape heterogeneity’ approach, where different cover types are classified by their physical characteristics without reference to a particular species or species group, with a ‘functional landscape heterogeneity’ framework in which heterogeneity is based on the expected functions (e.g., provision of food, nesting sites, dispersal routes) provided by that heterogeneity to the species or species group(s) of interest. This approach should produce models of higher predictive power, with resulting improved conservation and management strategies. Although in its infancy, the functional landscape framework could also shed some much needed light into the importance of the role played by the tissue microhabitat heterogeneity on tumor development and metastasis by moving the focus from definitions of tissue microhabitat constituents based on cell type to definitions based on the functions they provide to the tumor community.

It is important to note that while we emphasize strong similarities between the responses of animal and cancer cell populations to landscape structure, this was done by the way of analogy. An important next step would be to ascertain the actual degree of similarity between these two systems. To demonstrate that predictions derived from the landscape ecology literature are reliable, it will be important to assess the proportion of scenarios where tumor development fails to follow them; for example, the responses of cancer cell populations to blood vessels in the tissue microhabitat might be best predicted by animal population responses to rivers, to roads, to powerline cuts, or to none of these. Furthermore, it would be important to expand this framework to liquid tumors, which are notably different than the solid tumors discussed herein, perhaps through the use of the riverscape literature.

In sum, if we are to realistically meet the enormous technical and conceptual challenges of curing and preventing cancer, interdisciplinary collaborations among oncologists, biochemists, molecular and cellular biologists, evolutionists, and landscape ecologists are an important and crucial step forward. Such collaborations should not only benefit cancer research, but could also lead to new perspectives into macroecology as well.
